# Self-Repair in Cacti Branches: Comparative Analyses of Their Morphology, Anatomy, and Biomechanics

**DOI:** 10.3390/ijms21134630

**Published:** 2020-06-29

**Authors:** Max D. Mylo, Friederike Krüger, Thomas Speck, Olga Speck

**Affiliations:** 1 Plant Biomechanics Group, Botanic Garden, Faculty of Biology, University of Freiburg, Schänzlestraße 1, D-79104 Freiburg, Germany; friederike.krueger@imtek.uni-freiburg.de (F.K.); thomas.speck@biologie.uni-freiburg.de (T.S.); olga.speck@biologie.uni-freiburg.de (O.S.); 2 Cluster of Excellence *liv*MatS @ FIT—Freiburg Center for Interactive Materials and Bioinspired Technologies, University of Freiburg, Georges-Köhler-Allee 105, D-79110 Freiburg, Germany; 3 Laboratory for Chemistry and Physics of Interfaces (CPI) Department of Microsystems Engineering—IMTEK, University of Freiburg, Georges-Köhler-Allee 103, D-79110 Freiburg, Germany; 4 Freiburg Materials Research Center (FMF), University of Freiburg, Stefan-Meier-Straße 21, D-79104 Freiburg, Germany

**Keywords:** *Opuntia ficus-indica*, *Cylindropuntia bigelovii*, Opuntioideae, damage management, self-healing, wounding effect, healing effect, bending test, 3D printing

## Abstract

Damage-repair is particularly important for the maintenance of the water-storing abilities of succulent plants such as cacti. Comparative morphological, anatomical, and biomechanical analyses of self-repair were performed on artificially wounded branches of *Opuntia* *ficus-indica* and *Cylindropuntia bigelovii*. Macroscopic observations, contrast staining, and lignin-proof staining were used to investigate morphological and anatomical responses after wounding at various time intervals. Two-point bending tests were repeatedly performed on the same branches under unwounded, freshly wounded, and healed conditions by using customized 3D-printed clamping jaws. Morphologically, both species showed a rolling-in of the wound edges, but no mucilage discharge. Anatomically, ligno-suberized peridermal layers developed that covered the wound region, and new parenchyma cells formed, especially in *O. ficus-indica*. In all samples, the wounding effect directly after damage caused a decrease between 18% and 37% in all the tested mechanical parameters, whereas a positive healing effect after 21 days was only found for *C. bigelovii*. Based on our data, we hypothesize a high selection pressure on the restoration of structural integrity in the wound area, with a focus on the development of efficient water-retaining mechanisms, whereas the concept of “sufficient is good enough” seems to apply for the restoration of the mechanical properties.

## 1. Introduction

Damage control is a crucial concept in the plant kingdom as it can preserve the functionality of a plant and ultimately ensure its survival. In materials systems, damage control is often used as an umbrella term, covering the two aspects of damage prevention and damage management in the sense of complementary design concepts [1]. Plants can deal with existing damage in various ways, such as shedding old, injured, or no longer needed organs (so-called “abscission”) [2,3] or by using their self-repair ability to restore injured tissues and organs [4,5]. The latter is generally divided into an initial, rapid, self-sealing phase that functionally repairs the wound, protects the plant against serious water loss and pathogen invasion (bacteria, fungi, spores, etc.), and impedes further fissure propagation. The driving forces involved are mostly physical reactions such as the swelling of cells, the melting of waxes, and the deformation of wound tissues or entire organs [6]. Subsequently, during the long-lasting self-healing phase, the damage is structurally repaired by a process characterized by cell division and tissue regeneration, both of which restore (at least partly) mechanical properties and functions [7]. In latex-bearing plants, the chemical coagulation of latex immediately after injuries has been shown also to play a role in the recovery of tensile strength [8].

Self-repair plays a particularly important role in the group of succulent plants, because of their special adaptations in terms of water storage. A functional principle of self-sealing that can be found in various succulent species is the rolling-in of wound edges, driven by a passive change of turgor pressure. The epidermis and underlying cells lose a greater proportion of water than the cuticle, resulting in a functional two-layer laminate structure that causes wound closure while releasing mechanical pre-stresses and pre-strains [9,10]. The spectrum of self-healing responses is broad and varies between species depending on, for example, their bauplan. Reactions can be of a chemical (e.g., coagulation of latex), biochemical (e.g., the formation of a ligno-suberized boundary layer), or biological (e.g., development of a wound periderm) character [1].

The cactus family is among the best-known representatives of succulents, not least because of the specific growth form of cacti. They possess a wide range of water-storing adaptations that are noticeable at various levels: morphologically, they exhibit large volume-to-surface ratios in their aboveground parts (stems), very shallow and long root systems with cork layers, and the ability to produce rapidly a large number of hair roots after rainfall; anatomically, their stems are characterized by thickened outer epidermal and cuticular layers and a large proportion of water-storing parenchyma; physiologically, their metabolism (CAM photosynthesis) allows them to keep their stomata closed during the day; biochemically, they produce a polysaccharide mucilage, which has a tremendous water-storing capacity [11,12]. Initial anatomical experiments on the self-repair ability of cacti were published more than a century ago [13]; a successive periderm formation and newly built vascular bundles appeared in a V-shaped wound area after artificial incisions were made in branches of species from the tribes of Opuntioideae and Cylindropuntioideae. At a biomechanical level, bending tests have been conducted to investigate the mechanical properties of entire cacti stems [14,15] and of single tissues such as xylem strands [16]. Compression and tensile tests have been carried out on individual tissues as a basis for the finite element analyses of the branch-stem junctions of various columnar cacti [17]. In addition, branch–branch junctions have been studied in various species of Opuntieae [18,19] and Cylindropuntieae [20]. Two representatives of these tribes are *Opuntia ficus-indica* (L.) Mill and *Cylindropuntia bigelovii* (Engelm.) F.M. Knuth both belonging to the subfamily of Opuntioideae (family of Cactaceae). Whereas *O. ficus-indica*, original presumably native to Mexico, has spread over large parts of the globe in recent decades and can be found on all continents, mostly close to the coast [21], *C. bigelovii* can only be found in desert regions in the southwest of the USA and the northwest of Mexico [22]. The ability to resist mechanical failure in the junctions is considerably more pronounced in *Opuntia ficus-indica* [23] compared with *Cylindropuntia bigelovii* [20]. This can be directly linked to their principal reproduction mode: sexual reproduction via fruits in *O. ficus-indica* and vegetative reproduction via detached branches in *C. bigelovii* [11,24,25]. Biomechanical self-repair analyses of cacti, however, have not previously been carried out to the knowledge of the authors.

Since plants are multifunctional units, quantification of their repair process is neither trivial nor all-embracing. In order to perform a self-repair analysis that is as meaningful and comparable as possible, various approaches have been proposed to calculate healing efficiencies for single parameters, taking measurements in the unwounded and healed state [26,27] or, additionally, in the wounded state [28] into account. As healing efficiencies do not possess a unit, they are suited for comparisons between biological species and between biological and (bioinspired/biomimetic) technical systems.

We have undertaken a comparative morphological, anatomical, and biomechanical analysis of wound reactions in cacti branches of the closely related species *O. ficus-indica* and *C. bigelovii*. Following exclusively mechanical damage in terms of a ring incision, self-sealing and self-healing have been examined over a time span of up to 31 days, and self-repair effects have been compared with unwounded and freshly injured samples. In this context, three main aspects have been examined: (i) morphological and anatomical analyses providing information on the restoration of structural integrity, combined with (ii) biomechanical testing of unwounded, freshly wounded, and healed branches by using newly developed, 3D-printed clamping jaws; and (iii) the wounding effect directly after wounding ($WE_{0}$) and after a healing period of 21 days ($WE_{21}$), as a measure for the influence of the damage on certain mechanical parameters, and the healing effect as a measure for the restoration of the mechanical integrity after the three-week healing period ($HE_{21}$).

## 2. Results

### 2.1. Macroscopic Observations

**Figure 1:**
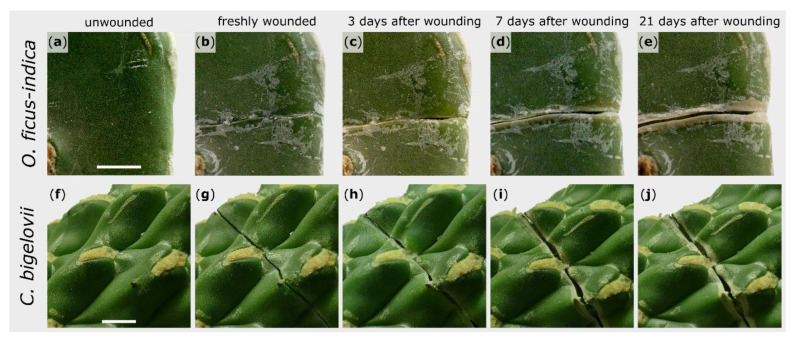
Morphological wound analysis of *Opuntia ficus-indica* (upper images) and *Cylindropuntia bigelovii* (lower images). The same section of one branch per species is presented in an unwounded condition (**a**,**f**), immediately after wounding (**b**,**g**), and after a healing phase of 3 days (**c**,**h**), 7 days (**d**,**i**), and 21 days (**e**,**j**). Scale bars = 5 mm.

The external sealing and healing processes of exemplary branches after artificial wounding were macroscopically recorded over a repair phase of 21 days. The wound regions shown are representative for all observed wounds of the respective species. The wound edges of *O. ficus-indica* remained in contact directly after wounding (Figure 1b), whereas a small gap was found immediately after wounding in *C. bigelovii* (Figure 1g). Subsequently, a rolling-in of the wound edges became visible for both species (Figure 1c,h). After about a week, white tissue comprising dead cell layers occurred parallel to the wound (Figure 1d,i). In *C. bigelovii*, this was accompanied by a simultaneous opening of the wound, whereas in *O. ficus-indica*, a pronounced opening of the wound was only apparent after two to three weeks (Figure 1e). Excessive secretion of mucilage could not be observed in either species at any time.

### 2.2. Microscopic Observations

**Figure 2:**
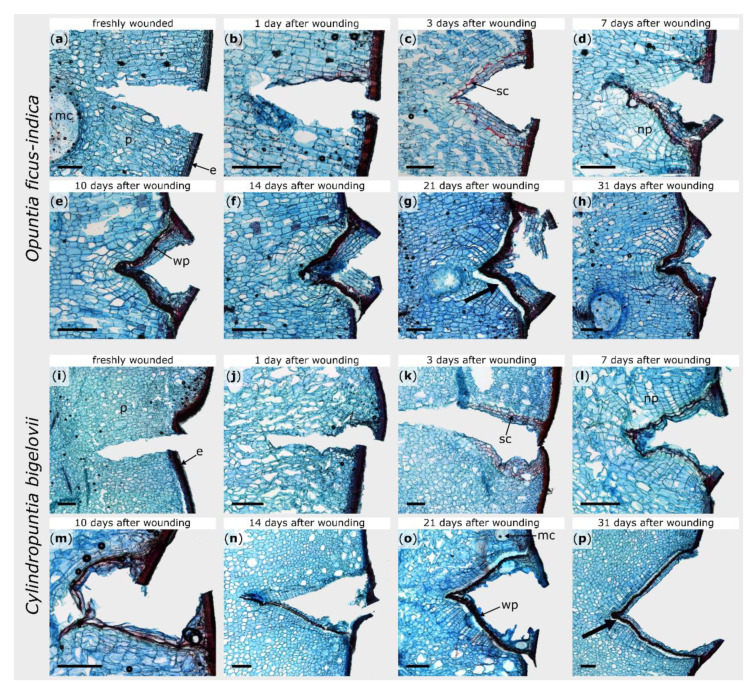
Tangential sections of wound areas after artificial ring incision in *Opuntia ficus-indica* (**a**–**h**) and *Cylindropuntia bigelovii* (**i**–**p**), stained with safranin O and Astra blue. The sections were microscopically examined directly after wounding (**a**,**i**), after a healing period of 1 day (**b**,**j**), 3 days (**c**,**k**), 7 days (**d**,**l**), 10 days (**e**,**m**), 14 days (**f**,**n**), 21 days (**g**,**o**), and 31 days (**h**,**p**). Cellulose cell walls appear blue, ligno-suberized cell walls appear red. e: epidermal and hypodermal layers, mc: mucilage cell, np: newly formed parenchyma, p: parenchyma, sc: suberized cells, wp: wound periderm. The bold arrows indicate exemplarily the areas where the phellogen has detached from the phelloderm caused by the mechanical stresses during the preparation of the section. Scale bars = 500 µm.

General branch anatomy and cellular repair processes after injury were documented by using stained microscopic thin sections. The terminal branches of both species possess an epidermis, covered by a thick cuticle, followed by three to five layers of closely packed hypodermal cells. For *O. ficus-indica*, the hypodermis consists of a single outer layer with solitary druses (groups of crystals) and multiple layers of collenchyma cells [29]. The parenchymatous cortex cells are arranged in a palisade pattern, although this pattern is much more pronounced in *O. ficus-indica*, whereas the cells appear more unordered in *C. bigelovii*. Vascular bundles are located between the cortex and water-storing parenchymatous pith cells (Figure 2). No injured vascular bundles of this central ring were visible in any of the prepared sections.

Within the first hours after wounding, no cellular change could be observed in either species (Figure 2b,j). Between day one and day three, the formation of lipophilic substances in the cell walls of the parenchyma near the injury became visible in both plants (Figure 2c,k). Since no lignin-sensitive staining occurred (see Supplement Figure S1c,k), the lipophilic substance can be assumed to be suberin. In *O. ficus-indica*, this accumulation was particularly pronounced in one cell layer (three to six cell layers beneath the wound surface) and covered the entire wound region, whereas, in *C. bigelovii*, it comprised several cell layers and extended from the epidermis into the interior of the branch. It took about one week until the entire wound of *C. bigelovii* was surrounded by newly formed cells with thickened cell walls (about two to four cell layers underneath the wound; Figure 2l). Lignin formation in *O. ficus-indica* began at day seven and then increases successively (Supplement Figure S1d–h). In *C. bigelovii*, lignification became visible from day 10 on and increased more slowly and was less pronounced than in *O. ficus-indica* (Supplement Figure S1m–p).

Furthermore, in both species, newly formed parenchyma cells were produced beneath the ligno-suberized layers (Figure 2d,l). In *O. ficus-indica*, these cell divisions become more pronounced over the next few days and weeks and led to an outgrowing of the wound notch, whereas the v-shape of the wound remained in *C. bigelovii* (Figure 2e–h,m–p). After 31 days, the wound was completely sealed by wound periderm in both species. However, from day 14 on, the connection between the phellogen and phelloderm was very delicate and tore off easily when microscopic sections were prepared (see bold arrows in Figure 2).

### 2.3. Biomechanics

#### 2.3.1. Bilinear Behavior

**Figure 3:**
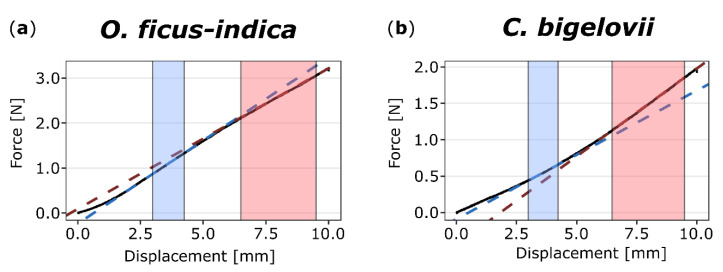
Exemplary force-displacement diagrams of two-point bending tests on (unwounded) branches of *Opuntia ficus-indica* (**a**) and *Cylindropuntia bigelovii* (**b**). The sections (colored background) and linear regression lines (colored dashed lines) of the first (from 3.0 to 4.25 mm, blue) and second (from 6.5 to 9.5 mm, red) linear ranges are highlighted. For *O. ficus-indica*, a decrease of slope from the first to the second range is visible, whereas for *C. bigelovii*, the value increases.

Force-displacement diagrams of the bending tests showed the bilinear behavior of both *O. ficus-indica* and *C. bigelovii* (Figure 3). The coefficients of determination (*R*^2^) for the respective linear regression lines indicated that the selected ranges were suitable for all samples (first linear range: *R*^2^ for all samples > 0.97; second linear range: *R*^2^ for all samples > 0.95). For both species, the calculated bending stiffness ratios (cf. Equation (2)) were not significantly different between the unwounded, freshly wounded, and healed states. Bending stiffness decreased about 7% from the first to second linear part in *O. ficus-indica*, resulting in ratios very significantly smaller than one, whereas in *C. bigelovii*, bending stiffness increased about 14% from the first to the second linear range, resulting in ratios highly significantly larger than one. These trends of bilinear stiffening or softening were observed for the individual states and for the pooled data of all states (Table 1).

#### 2.3.2. Bending Stiffness

In order to achieve a minimum of 14 samples for each species, branches from different plants had to be examined. Table 2 shows the absolute values of bending stiffness (cf. Equation (2)) of the two individual plants A and B of *O. ficus-indica* and the three plants C–E of *C. bigelovii.* For better comparability, two-point bending tests were performed on the same branches in the unwounded, freshly wounded, and healed (21 days after wounding) state.

**Figure 4:**
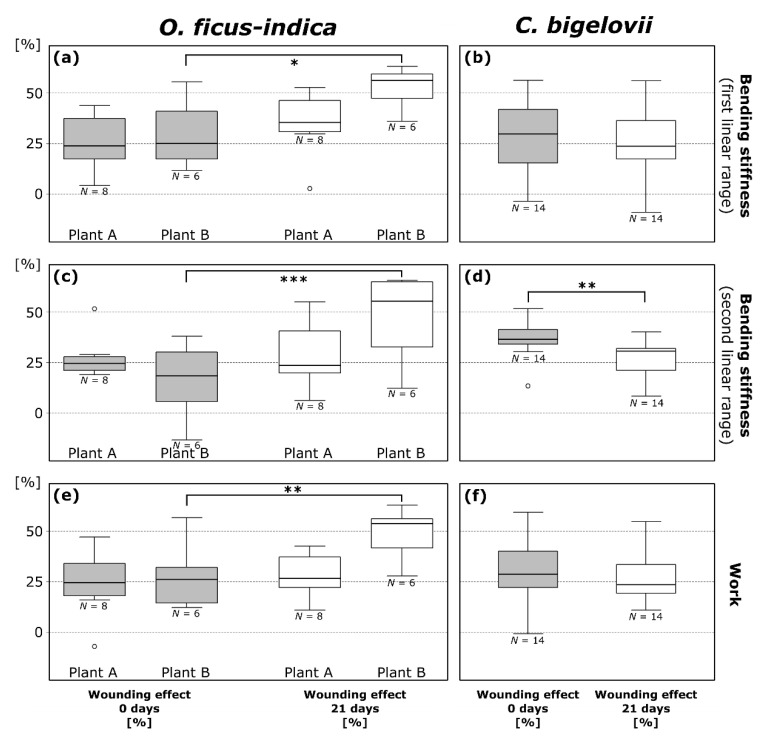
Wounding effects (cf. Equation (3)) directly after ring incision (gray box-plots; calculated from the respective values in the wounded and unwounded state) and after a healing period of 21 days (white box-plots; calculated from the respective values in the healed and unwounded state) of bending stiffness in the first (**a**,**b**) and second (**c**,**d**) linear range and work (**e**,**f**) determined from repeated bending tests on branches of *Opuntia ficus-indica* and *Cylindropuntia bigelovii*. All data points outside the range of 1.5 IQR are considered outliers and are represented by an “o”. The values of the three plants of *C. bigelovii* are presented as pooled since no significant difference was measured for any of the parameters. * *p* ≤ 0.05, ** *p* ≤ 0.01, *** *p* ≤ 0.001.

For a straightforward overview and the possibility of comparing all plants with each other, a normalization of the values in relation to the unwounded status was performed. The wounding effect (WE; cf. Equation (3)) is a measure of the degree of mechanical damage (Figure 4), whereas the healing effect (HE; cf. Equation (4)) is a measure for the mechanical recovery after a healing period (Table 3).

Wounding effects directly after ring incision ($WE_{0}$) as calculated from the values of bending stiffness do not differ significantly between the first and second linear range within the species with the exception of plant E (*t* = 3.871, df = 4, *p* = 0.018; paired *t*-test). Mean values, combined for values from the first and second linear range, were 24.9 ± 14.2% for *O. ficus-indica* (plant A: 26.5 ± 11.2%; plant B: 22.8 ± 17.2%) and 33.1 ± 14.6% for *C. bigelovii* (plant C: 35.8 ± 15.7%; plant D: 29.5 ± 17.5%; plant E: 1. linear range: 28.0 ± 8.5%, 2. linear range: 38.9 ± 6.4%). $WE_{0}$ within the first or the second linear range did not differ significantly in a comparison of plants A and B of *O. ficus-indica* and plants C, D, and E of *C. bigelovii*. A detailed overview of wounding and healing effect data can be found in the Supplementary Table S1.

Wounding effects after a healing period of 21 days ($WE_{21}$) calculated from the values of bending stiffness did not differ significantly between the first and second linear range within the species. Mean values are 39.9 ± 17.8% for *O. ficus-indica* (plant A: 31.9 ± 15.2%; plant B: 50.4 ± 15.3%) and 27.7 ± 13.7% for *C. bigelovii* (plant C: 23.7 ± 16.9%; plant D: 27.2 ± 9.8%; plant E: 31.5 ± 11.4%). When comparing the three tested plants C, D, and E of *C. bigelovii,* $WE_{21}$ did not differ significantly in the first or second linear range. For *O. ficus-indica*, however, $WE_{21}$ differed significantly between plants A and B exclusively in the first linear range (*p* = 0.022). Significant increases from $WE_{0}$ to $WE_{21}$ were found for plant B of *O. ficus-indica* with regard to bending stiffness in the first (*p* = 0.017; Figure 4a) and second (*p* = 0.00076; Figure 4c) linear range. $WE_{0}$ and $WE_{21}$ did not differ significantly for plant A of *O. ficus-indica*. A very significant decrease from $WE_{0}$ to $WE_{21}$ was found for pooled data of *C. bigelovii* in the second linear range (*t* = 3.321, df = 13, *p* = 0.0055; paired *t*-test; Figure 4d).

Positive values of the healing effect after a three-week period ($HE_{21}$) indicate a mechanical healing effect, whereas negative values point to a further deterioration of the mechanical stability during the three-week healing period (Table 3). In the first linear range, $HE_{21}$ of plant A and B of *O. ficus-indica* did not differ significantly. $HE_{21}$ of plant B and pooled data were significantly lower than zero. In the second linear range, values for plant A and B differed very significantly (*p* = 0.0038), with the values for plant B being significantly lower than zero. No significant difference was found for $HE_{21}$ values between the three plants of *C. bigelovii* for the first linear range (ANOVA: *p* = 0.040, Tukey’s test: all *p* > 0.055), with only plant A being significantly higher than zero, or for the second linear range, with all individual and the pooled data being significantly higher than zero. Further information on the detailed statistical results can be found in Table A1 and Table A2.

#### 2.3.3. Work

The absolute values of work of the individual plants are presented in Table 2. Wounding effects (WE, cf. Equation (3)) and healing effects (HE, cf. Equation (4)) were also calculated for work represented by the discrete integral under the force-displacement curve. Plants A and B of *O. ficus-indica* did not differ significantly in terms of $WE_{0}$ and $HE_{21}$ but showed very significant differences in comparisons of the $WE_{21}$ of work (*p* = 0.0078). For plant A, no significant differences between $WE_{0}$ and $WE_{21}$ could be found (mean $HE_{21}$ = −4.0 ± 17.6%, no significant difference to zero), whereas plant B showed a very significant decrease in work from $WE_{0}$ to $WE_{21}$ (*p* = 0.0062; Figure 4e) and from $HE_{21}$ (21.3 ± 10.5%) to zero. On comparing plants C, D, and E of *C. bigelovii*, no significant difference between the $WE_{0}$ and $WE_{21}$ values was found, and the data were pooled. Furthermore, no significant increase from $WE_{0}$ to $WE_{21}$ was found for the pooled data of *C. bigelovii* (Figure 4f). The same applied to the differences between $HE_{21}$ (2.1 ± 10.9%) and their difference from zero (Table 3). Further information on detailed statistical results can be found in Table A1 and Table A2.

#### 2.3.4. Bending Elastic Modulus

The bending elastic modulus (E) of unwounded branches was calculated according to Equation (5). Mean values of E for *O. ficus-indica* were 6.4 ± 1.8 MPa for plant A (axial second moment of area I: 2445 ± 834 mm^4^) and 4.0 ± 1.6 MPa for plant B (I: 4464 ± 2101 mm^4^). Values for *C. bigelovii* were 0.54 ± 0.14 MPa for plant C (I: 24,041 ± 4138 mm^4^), 0.60 ± 0.04 MPa for plant D (I: 30,662 ± 6688 mm^4^) and 0.56 ± 0.16 MPa for plant E (I: 31,256 ± 3701 mm^4^). No significant differences were found between the two plants of *O. ficus-indica* (t = 0.80, df = 5.68, *p* = 0.46; Welch’s two-sample t-test), or between the plants of *C. bigelovii* (Df = 2, Sum Sq = 0.0095, Mean Sq = 0.0047, F = 0.23, *p* = 0.80). However, highly significant differences were found between the pooled values of the two species (*t* = 9.92, df = 14.16, *p* < 0.001; Welch’s two-sample *t*-test).

#### 2.3.5. Synopsis of Biomechanical Performance

In summary, no positive mechanical healing effects on bending stiffness were found for *O. ficus-indica*, whereas these occurred particularly in the second linear range for *C. bigelovii*. In general, negative healing effects on work were observed for *O. ficus-indica*, while there was no significant effect for *C. bigelovii*. During the healing period, the bending stiffness of plant A decreased moderately and the work remains almost unchanged. The bending stiffness and the related work of plant B dropped significantly. The bending stiffness of plant C decreased markedly, whereas the work increased slightly. While the bending stiffness of plant D decreased moderately in the first linear range, bending stiffness in the second linear range and the work increased. While the bending stiffness of plant E decreased in the first linear range, it increased significantly in the second linear range. The work of plant E decreased.

Mean values of the bending elastic modulus of *O. ficus-indica* are one order of magnitude higher than those of *C. bigelovii.* The median bending elastic modulus of plant B is 1.9 times higher than that of plant A, whereas the values of plant C, D, and E differ by a maximum factor of 1.1.

#### 2.3.6. Relative Water Content

For *O. ficus-indica*, values of relative water content (RWC) did not differ significantly between plants. Wounded branches (median value: 95.8%) had a very significantly lower RWC than control branches (99.0%; Wilcoxon rank-sum test, *p* = 0.0058). Values exceeded 100% for one tested and seven untested branches, indicating an unknown influence of mucilage removal. RWC values were not significantly different between plants for *C. bigelovii*. No significant difference could be found between wounded (81.9%) and control branches (79.6%).

## 3. Discussion

On the one hand, the self-repair of plants after a mechanical injury can be divided according to the time aspect into a rapid sealing phase followed by a longer-lasting healing phase. On the other hand, self-repair can also be subdivided according to the self-repair effects on the structural integrity in terms of morphological and anatomical wound reactions and on mechanical integrity in the sense of restoring the original mechanical performance of selected parameters after the healing of an injury. Since there are some morphological-anatomical similarities between wound reactions and the abscission of plant organs, this aspect is also briefly touched upon.

### 3.1. Morphological and Anatomical Repair-Effects: Restoration of Structural Integrity

*O. ficus-indica* and *C. bigelovii*, as representatives of the Opuntioideae, the second-largest subfamily of cacti, have evolved special adaptations for improved water storage because of their distribution in (semi-)arid habitats. The preservation of stored water is crucial for the survival of these species. Therefore, injuries to the outer tissue involve not only the danger of pathogen invasion and loss of mechanical performance but also the risk of enormous water loss. A high selection pressure on the restoration of structural integrity through morphological and anatomical wound reactions can thus be assumed.

The observed rolling-in of the wound edges as a fast self-sealing mechanism is a recurring pattern in succulent plants [9,10]. It can be attributed to the different dehydration properties of the cuticle compared with the epidermis and hypodermis leading to superficial wound closure, even if a gap remains inside. According to Anandan et al. [9], the immediate closure of the wound in *O. ficus-indica* indicates the greater compressive pre-stresses in a radial direction than tensile pre-stresses in a longitudinal direction. In *C. bigelovii*, an equilibrium of pre-stresses seems to be present, as neither direct closing nor direct opening of the wound is apparent. A superficial accumulation of mucilage in the wound region after the incision, as is suspected for cacti [9,30], was not detectable in any of the examined samples. The anatomical analyses in this study, however, allow no statement to be made about the possible sealing of wound areas with mucilage since the production of sections thin enough for anatomical investigation could only be achieved by the removal of the mucilage with bleach prior to staining. Moreover, the macroscopic observations revealed no discharge of mucilage within the wound region.

After one to three days, a boundary layer of lipophilic substances forms near the wound surfaces; this layer most likely consists initially only of suberin and later additionally of lignin. The formation of a single or multicellular cell layer with ligno-suberized cell walls is a common repair mechanism that occurs in a variety of other succulent plant species [9,13].

In both species, the newly formed wound periderm completely covers the wound after healing. Between the two species, only small differences were found regarding the temporal pattern of the formation of the wound periderm; they differ mainly in structural details (Figure 2 and Figure S1). With regard to the time scales and the mechanisms of wound reaction, our results strengthen the early findings of Coutant [13] and suggest a comparable wound reaction pattern in the different species of Opuntioideae, as documented in this work, for the first time, with images at a cellular level. After a healing phase of 14 days, a clear weak spot remains in the wound region of both species: the connection between the phellogen and phelloderm is sufficient to restore structural integrity but is very fragile under mechanical stress. Hence, the previously existing mechanical integrity does not seem to be fully restored at these sites. This weak connection thus also affects the mechanical properties of the entire branch.

Ligno-suberized boundary layers and periderm formation described above in the context of wound healing are also regularly found in abscission zones [2]. These morphological-anatomical characteristics are on the one hand prerequisites for the shedding of fruits during sexual reproduction as found in *O. ficus-indica*. On the other hand, as in the case of *C. bigelovii*, they are necessary for the abscission of branches allowing for vegetative reproduction. Apart from these structural and functional similarities, however, differences can also be found, particularly in the chronological order of the phenomena that occur. If we hypothesized that the abscission zone is a “planned wound”, the preparatory wound reaction would be a controlled reduction of structural and mechanical integrity resulting in a subsequent separation of biological materials systems.

### 3.2. Biomechanical Repair-Effects: Restoration of Mechanical Integrity

Artificial ring incision of the branches allowed us to obtain median $WE_{0}$ values ranging between 18% and 37% for both species and for all three tested mechanical parameters (Figure 4). Therefore, we consider that the type and extent of wounding were chosen and executed in a reliable and comparable manner. In 3 of the 84 determined values (coming from two samples of *O. ficus-indica* and one sample of *C. bigelovii*), a $WE_{0}$ under 0% was measured (see outlier in Figure 4 and Supplement Table S1). An explanation for these unexpected $WE_{0}$ values ranging between −1% and −13% could be spontaneous unobserved rapid wound reactions (e.g., the influence of mucilage release; the interlocking of the wound surfaces” as these are two different aspects).

Both species showed a bilinear force-displacement curve during bending, resulting in a changing bending stiffness (cf. Figure 3). Such biphasic behavior has been observed several times in biomechanical studies on plants and algae (e.g., [31,32,33,34,35]) and for sandwich-structured composite materials [36] that the examined cacti resemble in their simplified structure (epidermis and vascular tissues as face sheets and cortex and pith as core tissue). However, in *O. ficus-indica,* we observed strain softening, whereas branches of *C. bigelovii* showed strain hardening (Table 1). In the first linear phase, the structure of the sample generally aligns itself according to the applied mechanical stresses. The net-like vascular bundles may also play an important role during alignment. In the second linear phase, the tissues involved are increasingly subjected to the respective tensile or compressive stresses. The differences in the bilinear behavior of *O. ficus-indica* and *C. bigelovii* can be partly explained by the branch geometry. In the case of *C. bigelovii*, increasing deflection might lead to an initial straightening of the undulating epidermis on the tension side of the branch and therefore to an alignment of the structure with the force flow. In contrast, *O. ficus-indica* has flat cladodes with no pronounced surface irregularities, and so, during initial force application, unfolding events on the tensile side can be neglected, and the forces can thus be transferred faster to the materials system. The stiffness softening of *O. ficus-indica* might be explained by the elastic buckling of the parenchyma under higher bending load [37]. Since strain softening and strain hardening are present in the unwounded, wounded, and healed states (cf. Table 1), they also seem to be a characteristic of the respective materials system. In both species, the strain-softening or strain-hardening is more pronounced in the unwounded specimens with an intact epidermis and hypodermis compared with freshly wounded samples having an intersected hypodermis. In *O. ficus-indica*, the bending stiffness ratio of the healed specimens does not differ from the freshly wounded specimens. This is probably because the wound periderm is only weakly attached to the hypodermis and breaks off at low mechanical stresses. In contrast, the bending stiffness ratio of *C. bigelovii* increases to the value of the unwounded samples. This result strongly suggests that, during wound healing in *C. bigelovii*, the structural integrity and consequently the mechanical integrity are restored more successfully than in *O. ficus-indica*.

Niklas et al. [15] carried out bending tests on intact stems of *Stenocereus eruca* and *S. gummosus* in order to determine their bending stiffness and bending elastic modulus. They found a mean bending stiffness of 0.81 MPa for *S. eruca* and 0.14 MPa for *S. gummosus*. The mean values of *S. eruca* are about 50 to 60 times and those of *S. gummosa* about 10 times higher than the mean values of the bending stiffness found for *O. ficus-indica* and *C. bigelovii*, respectively (cf. Table 2).

Calculation of the bending elastic modulus of (unwounded) branches resulted in mean values of 5.52 ± 1.76 MPa and 0.57 ± 0.13 MPa for *O. ficus-indica* and *C. bigelovii*, respectively. Although the determination of the axial second moment of area was difficult, these values are of the same order of magnitude as the mean values of the bending elastic moduli of 0.92 MPa for *Stenocereus eruca* and 2.11 MPa for *S. gummosus* [15]. The higher values for *O. ficus-indica* can be explained by its thickened epidermis, hypodermis, and cuticle layers; these tissues can vary enormously between the species of Opuntieae and Cylindropuntieae [29] and with regard to the various types of vessels present, as cacti species possess a diverse, sometimes delayed, the phase transition from juvenile wide-band tracheids (fibreless, flexible cells) to adult stiffer fibrous wood [38].

According to the bending tests of Nobel and Meyer [16], the branch stiffness in *O. ficus-indica* is almost twice as high as the stiffness of their branch-branch junctions. Due to the very fragile junctions of *C. bigelovii* [18], we might expect that this disparity is even more pronounced in the latter species. A wound-related loss of mechanical stability of a branch by 18% to 37% might therefore not make it the weakest link in the chain of individual branches of the overall structure. Thus, the complete recovery of mechanical properties of a wounded branch might be “less important” for the long-term survival of the plant than protection against a potentially lethal loss of water or a disastrous pathogen infection.

Surprisingly, the values for the bending stiffness in the second linear range of the samples of plant B of *O. ficus-indica* decreased significantly during the healing phase compared with the wounded state indicating that both structural integrity and mechanical integrity were further degraded during the healing process. One explanation for this response of *O. ficus-indica* might be associated with the observation that, beginning with day 14 after wounding, mucilage cells are frequently found in the immediate vicinity of the wounded areas. These mucilage cells are conspicuous by their sometimes large spatial requirements, which can change depending on the amount of water bound in the mucilage (cf. Figure 2g,h). Depending on their size and frequency in the wound region, the mucilage cells influence the overall mechanical performance of each branch, possibly explaining the differences between plants A and B, especially with regard to the recovery of the tested mechanical parameters.

Another parameter influencing the stiffness of a plant organ is its water status, which can be quantified by using the relative water content as a reliable measure [39,40,41]. Because of the enormous mucilage content of the plants and the immense ability of mucilage to store water [42], a slightly modified measuring procedure for the RWC had to be applied. We could not find a significant difference in water content between a control group of unwounded branches after a drought period of 21 days and the wounded branches for *C. bigelovii*, but we observed a very significant water loss in the wounded branches of *O. ficus-indica* (Wilcoxon rank-sum test, W = 186.5, *p* = 0.0023).

In order to obtain a comprehensive characterization of mechanical changes during and after self-repair, bending tests were conducted after various healing periods. Our preliminary tests and anatomical analyses indicate that the healing time of 21 days, which has also served as a useful interval in other studies [9,43,44], is a suitable time period for our experiments and additionally minimizes other influencing factors such as growth. The individualized 3D printed jaws used for the bending proved to be a suitable method for the performance of repeated bending tests under constant geometrical conditions. No epidermal damage was seen on the branches, either at the area of the jaws or at the collars. Imprints in the cuticle were evenly distributed over the whole contact area indicating adequate form closure. With the irregular geometry of the branches, the individualized jaws and collars fitted only in one place and did not appear to slip during the tests. These features indicate that our method represents an enhancement of mechanical tests on biological samples, especially for bending and tensile tests for which precise measurements are often hampered by slipping effects.

## 4. Materials and Methods

### 4.1. Plant Material and Treatment

**Figure 5:**
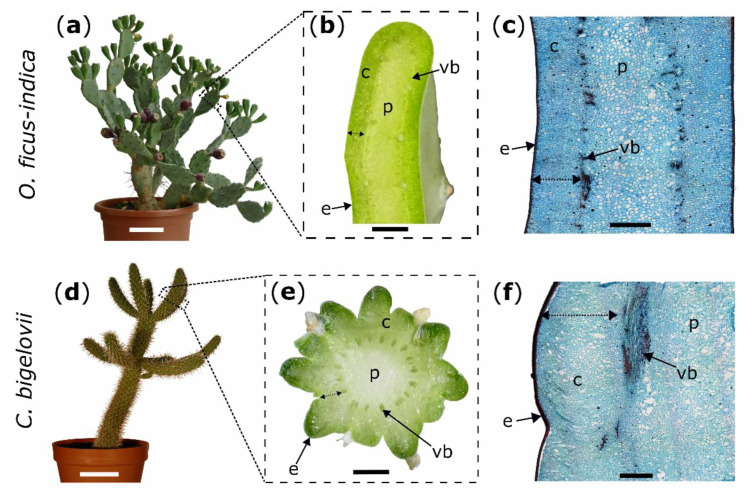
Experimental plants (left images) with macroscopic transverse sections (middle images) and microscopic tangential sections stained with safranin O and Astra blue (right images); *Opuntia ficus-indica:* (**a**–**c**); *Cylindropuntia bigelovii*: (**d–f**). Double arrows mark the measured cortex depth (**b**,**e**) and the intended depth of the ring incisions through the cortex down to the vascular bundles (**c**,**f**). c: cortex; e: epidermis with hypodermal layers; p: pith; vb: vascular bundle. Scale bars = 10 cm in (**a**,**d**), 5 mm in (**b**,**e**), and 2 mm in (**c**,**f**).

Experimental plants of *Opuntia ficus-indica* (L.) Mill. (Figure 5a) and *Cylindropuntia bigelovii* (Engelm.) F.M. Knuth were purchased from Kakteenland Steinfeld (Steinfeld, Germany) and cultivated in pots in a phytochamber at the University of Freiburg under constant conditions with a suitable day-night rhythm (day conditions: 8 a.m. to 7 p.m., 30 °C, 30% relative humidity, artificial lightning (about 6000–8000 cd·sr·m^−2^); night conditions: 8 p.m. to 7 a.m., 20 °C, 50% relative humidity, no illumination; conditions were adjusted during the remaining hours: between 7 p.m. and 8 p.m, and 7 a.m. and 8 p.m, respectively) six months prior to and during all experiments.

Only terminal branches without leaves or visible damage were selected in all experiments. A nail clipper was used to remove spines either on the entire tested branch (for biomechanical testing) or around the wounded area (for morphological and anatomical analyses). Fruits attached to the branches in *O. ficus-indica* were removed at least one month before testing. All tested plants were well watered three days before being wounded. Unless otherwise stated, no watering was performed during the healing phase.

### 4.2. Wounding

Artificial ring incisions were manually applied to the branches by using a scalpel. Wounds were located at 50% of total branch length for all morphological and anatomical analyses of the two species and bending tests in *C. bigelovii* and at 25% of total branch length (from base to apex) for bending tests in *O. ficus-indica*. To obtain comparable wounding effects in both species, epidermis, hypodermis, and cortex (summarized as outer tissue) were incised, whereas vascular bundles and the pith were allowed to remain intact (Figure 5c,f). Based on these pre-tests in which the outer tissue proportion of the total diameter (at its widest point) was measured, the relative cutting depths of the branch diameter were set to 10% for all experiments on *C. bigelovii*. Since the incisions were located at different total branch lengths with slightly different tissue distribution, the relative cuttings for *O. ficus-indica* were set to 20% for morphological and anatomical analyses and to 15% for biomechanical testing (Figure 5b,e).

### 4.3. Macroscopic Observations

Macroscopic wound monitoring was performed on one plant per species. Images of the wound region were recorded on an almost daily basis during a healing period of 31 days by using a camera (Lumix DMC-FZ1000, Panasonic Corporation, Kadoma, Japan) equipped with a macro lens (DHG Achromat Macro 200, 62 mm, Marumi Optical Co., Ltd., Tokyo, Japan).

### 4.4. Anatomy

Cubic samples were cut from one intact branch and the wounded areas of one freshly incised and ten healed branches (after healing periods of 5 h, 1 d, 2 d, 3 d, 5 d, 7 d, 10 d, 14 d, 21 d, and 31 d) for each species. All samples were covered with embedding medium (Tissue-Tek^®^ O.C.T.™ Compound, Sakura Finetek Europe B.V., Alphen aan den Rijn, Netherlands) and frozen for at least two hours in a floor-standing ECO cryostat (MEV, SLEE medical GmbH, Mainz, Germany). Samples were cut into tangential sections at a thickness of 75 µm by using the integrated microtome blade of the cryostat and were transferred to a 50% bleach solution (Eau du Javel, Floreal Haagen GmbH, Wadgassen, Germany) for 15 to 20 min to remove mucilage before being transferred into distilled water. For contrast staining, the cuttings were dipped into a safranin O solution (1 g safranin O in 100 mL distilled water) for three to five seconds, rinsed for 15 s with an acidic alcohol solution (0.5 mL of 30% hydrochloric acid with 100 mL of 70% ethanol), stained in an Astra blue solution (0.5 g Astra blue in 100 mL of 2% aqueous Tartaric acid) for 12 s and then washed in distilled water for 20 to 30 s. Images of stained sections were obtained via a stereomicroscope (Olympus BX61, Olympus Corporation, Tokyo, Japan) equipped with a microscope camera (DP71, Olympus Corporation, Tokyo, Japan) and Cell^P imaging software (Version 2.6, Olympus Soft Imaging Solutions GmbH, Münster, Germany).

A phloroglucinol solution (5 g phloroglucinol in 100 mL of 92% ethanol), to which about 10 to 20 drops of hydrochloric acid were added, was used as lignin-proof staining (a piece of paper towel consisting mainly of wood pulp dipped into the solution was used as the positive control). Images were taken with a light microscope (Primo Star, Carl Zeiss Microscopy GmbH, Jena, Germany) fitted with a microscope camera (AxioCam ERc 5s, Carl Zeiss Microscopy GmbH, Jena, Germany). Three samples of *C. bigelovii* (healing periods of 14, 21, and 31 days) were watered one day before anatomical analysis to increase cross-section stability.

### 4.5. Biomechanical Testing

**Figure 6:**
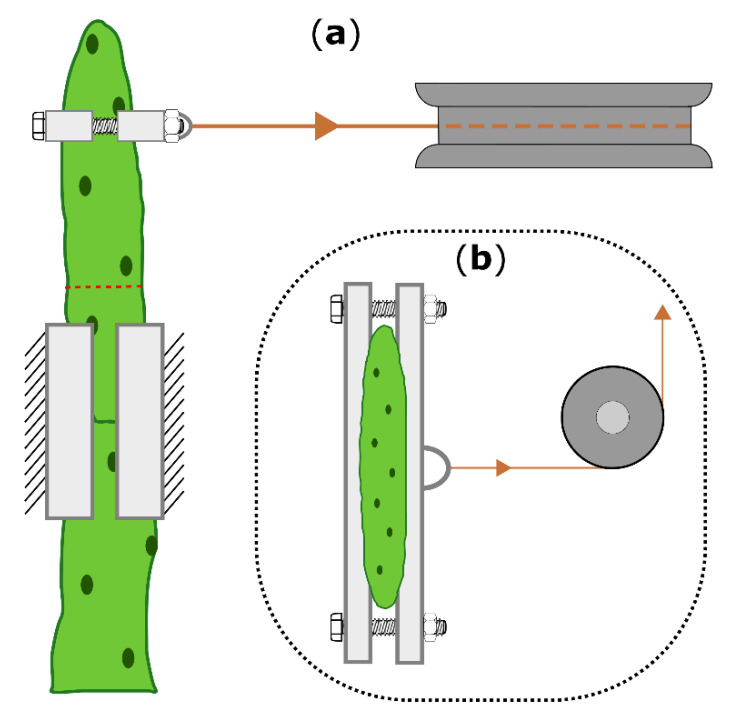
Schematic drawing of the two-point bending arrangement, shown for *Opuntia ficus-indica*. (**a**): Top view of the bending zone, including the 3D-printed clamps and collars (bright boxes), the pulley (dark gray), the Kevlar thread (orange), and the location of the ring incision (dashed red line). (**b**): Front view with attached collars and the pulley deflecting a vertical into a horizontal force. The universal testing machine above the pulley, which allows vertical force measurement, is not shown.

Two-point bending tests were conducted on the same branches in the unwounded (one day before ring incision), freshly wounded (within 15 min after incision), and healed (21 days after incision) states. Fourteen branches (length: 111 ± 19 mm, weight: 38 ± 16 g) from two plants (Plant A: 8 branches; plant B: 6 branches) of *O. ficus-indica* and fourteen branches (length: 175 ± 25 mm, weight: 88 ± 16 g) from three plants (Plant C and E: 5 branches; plant D: 4 branches) of *C. bigelovii* were bent to a deflection of 10 mm by using a universal testing machine (Inspekt mini, Hegewald & Peschke Meß- und Prüftechnik GmbH, Nossen, Germany) equipped with a 100 N load cell (KAP-S, A.S.T. Angewandte System Technik GmbH, Wolnzach, Germany). The perpendicular bending force was applied via collars embracing the branch, a Kevlar thread (0.7 mm, SÜDTX Vertrieb Technische Schnüre Jutta Lingen & ProTec Abspannseile, Füssen, Germany), and a pulley underneath the loading cell (Figure 6).

**Figure 7:**
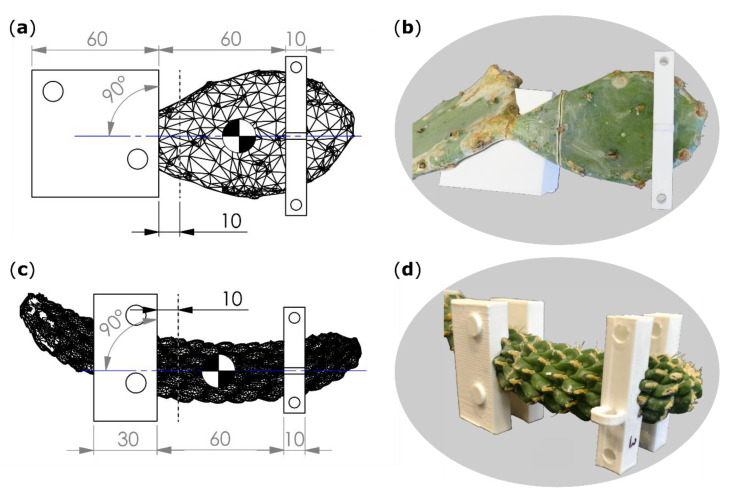
Technical drawings (**a**,**c**) including scanned branch models, artificial incision interface (gray dashed line), plane of bending (blue line), center of mass (quartered circle), and dimensions of clamping jaws (left rectangles) and collars (right rectangles). Images of the branches together with clamping jaws (for *O. ficus-indica* just one of the two halves) and collars (**b**,**d**). (**a**,**b**) *Opuntia ficus-indica*; (**c**,**d**) *Cylindropuntia bigelovii*. All length specifications are given in millimeters.

The bending speed was set to 1 mm/s, and the force and displacement values were acquired at 50 Hz. To minimize the influence of the branches’ own weight and to create comparable conditions for all tests, the plants with their pots were placed in a sandbox on a height-adjustable lift truck with the base-apex axis of the tested branch aligned parallel to the ground (cladode surfaces of *O. ficus-indica* orthogonally to the ground) and with its basal part fixed on a customized stand (Technical Workshop, Institute of Biology II/III, University of Freiburg, Germany). In order to generate the best possible form-fit and reproducible tests, individually constructed clamping jaws were used for each sample (Figure 7). For this purpose, terminal and sub-terminal branches were 3D scanned (Artec Spider, Algona GmbH, Stuttgart, Germany) before being tested. Several individual scans of one branch were merged in the scan software (“Autopilot mode”, Artec Studio 12 Professional, version 12.1.6.16, algona GmbH, Stuttgart, Germany), possible holes were automatically filled, and the resulting models were simplified with the fast mesh simplification algorithm to 10,000 nodes (for *O. ficus-indica*) or down to an accuracy of 0.1% (for *C. bigelovii*) and exported as STL files. The latter were imported into CAD software (SolidWorks 2019, Dassault Systèmes SolidWorks Corporation, Waltham, MA, USA) that automatically calculated the center of mass (COM) of the terminal branches.

Within the software, raw bending jaw models were positioned parallel to flattened branch surfaces of *O. ficus-indica* and parallel to symmetry planes of branches for *C. bigelovii* (Figure 7). The outer edge of the jaws was positioned 10 mm basal to the planned ring incision interface. Collar models were placed in a distance of 60 mm apical to the bending jaw models with their transmission points on an axis that was perpendicular to the raw bending jaw model and in line with the COM to avoid torque (Figure 7a,c). Form nests of the scanned branches were removed from the raw jaw and collar models, and the resulting individualized CAD models were printed on an extrusion 3D printer (Pro2 Plus, Raise 3D Technologies, Inc., Irvine, CA, USA) with a polylactic acid filament (Premium PLA, Formfutura BV, Nijmegen, Netherlands). Collars were fixed on the sample with two screws and built-in drill holes.

### 4.6. Wounding Effect and Healing Effect

The work required to deflect the branch by 10 mm was calculated as the discrete integral under the force-displacement curve by using the trapezoidal rule. Since the curve showed a biphasic linear behavior, bending stiffness (*EI*) was calculated for a first (from 3 to 4.25 mm deflection) and second (from 6.5 to 9.5 mm deflection) linear range by using the following equation:

(1) $$EI=\frac{L^{3}}{3*b}$$

where *L* is the length of the cantilever (60 mm for all presented experiments) and *b* the slope of the respective linear elastic range in the displacement-force diagram. The ratio of the two bending stiffnesses was calculated to quantify their relation as:

(2) $$Bending\;stiffness\;ratio=\frac{Bending\;stiffness\;of\;second\;linear\;range}{Bending\;stiffness\;of\;first\;linear\;range}$$

Resulting values for work and bending stiffness from the different states were used to calculate the wounding effect (WE) by using the following equation:

(3) $$WE\;\left(\%\right)=\frac{unwounded\;value-wounded\;value}{unwounded\;value}*100$$

The wounding effect is a measure for the degree of wounding on the mechanical integrity with reference to individual mechanical parameters. The unwounded value (uv) is measured before damage, the wounded values are measured either directly after wounding ($wv_{0}$) or after a healing period of 21 days ($wv_{21}$). WE = 0% if $uv=wv_{0}$, which reflects a fully intact and undamaged status or if $uv=wv_{21}$, which mirrors a fully healed status. WE = 100%, if wv=0, which reflects the fully damaged status. The formation of new material with different mechanical properties can lead to $wv_{21}>uv$ and thus to negative percentages of WE.

The healing effect (HE) after a healing period of 21 days is calculated by using Equation (4):

(4) $$HE_{21}\;\left(\%\right)=WE_{0}-WE_{21}=\frac{wv_{21}-wv_{0}}{uv}*100$$

with $WE_{0}$ being the wounding effect of freshly incised samples and $WE_{21}$ being the wounding effect 21 days after incision.

### 4.7. Bending Elastic Modulus

Calculation of the bending elastic modulus from the bending stiffness (EI) requires the axial second moment of area (*I*) of the entire sample. However, the determination of *I* is difficult because of the demanding geometry of the cactus branches. For this reason, values for *I* at the clamping jaw and at the collar of each branch were calculated from the 3D scans by using SolidWorks. The mean values of *I* were calculated as a simplification of the complex branch geometries in order to estimate the bending elastic modulus of the branch as a geometry-independent material property (based on the bending stiffness of first linear range). The elastic modulus (E) is calculated by using Equation (5):

(5) $$E=\frac{EI}{I}$$

### 4.8. Relative Water Content

The relative water content (RWC) of tested branches (all 15 branches of *O. ficus-indica* and 10 branches of *C. bigelovii*) was measured after bending tests were conducted in the healed condition. An equal number of untested (lateral) branches were concurrently harvested from the same plants if possible, resulting in 15 control branches for *O. ficus-indica* (from two plants) and 7 control branches for *C. bigelovii* (from two plants). For *O. ficus-indica*, three circular samples with a diameter of 12 mm were punched out of branches immediately above (two samples) and immediately below (one sample) the incision plane. For *C. bigelovii*, two slices with a thickness of about 2 mm were extracted, one 5 mm apically and one 5mm basally of the incision plane. Samples of untested branches were removed from similar positions. The fresh weight of all samples was taken immediately after cutting had been performed (all weight measurements were performed using an analytical balance: ABT 220-5DM, reproducibility: 0.1 mg, KERN & SOHN GmbH, Balingen-Frommern, Germany). All samples were kept in distilled water for two hours, and turgescent weight was measured after the careful removal of excessive water. Because of the immense water storing capacity of mucilage and the accompanying difficulty in the removal of excess water, all samples of *O. ficus-indica* were placed in a highly basic solution (MucoFlutol, MORPHISTO GmbH, Frankfurt am Main, Germany) for 10 to 15 min beforehand, in order to avoid mucilage discharge, and hence to minimize the influence of mucilage on fresh weight. After storage of the samples in a drying cabinet at 60 °C for at least 24 h, we measured their dry weight. RWC was calculated by using Equation (6). Mean RWC values of extracted samples were calculated per branch.

(6) $$\mathrm{RWC}\;\left(\%\right)=\frac{\mathrm{fresh}\;\mathrm{weight}-\mathrm{dry}\;\mathrm{weight}}{\mathrm{turgescent}\;\mathrm{weight}-\mathrm{dry}\;\mathrm{weight}}*100$$

### 4.9. Statistics

Raw data (see Supplemental Table S1) were recorded and analyzed with Microsoft^®^ Office EXCEL^®^ 2016. Data are either represented by mean values ± one standard deviation or shown as median values with respective interquartile ranges (IQR). Data processing, data visualization, and statistical analyses were performed with GNU R v.3.6.1 [45], including the packages *car* [46], *coin* [47], *DescTools* [48], *ggplot2* [49], *multcomp* [50], and *psych* [51]. Descriptive statistics were used to describe morphometric data, bending stiffness ratios, RWC values, and approximated values for the bending elastic modulus. Once the assumptions for normally distributed data (Shapiro-Wilk test; α = 0.05) and homoscedasticity (Levene test; α = 0.05) had been checked, datasets from the different plants of one species were tested for significant differences by using the Welch’s two-sample t-test (for *O. ficus-indica*, normally distributed data with equal variances), Wilcoxon rank-sum test (for *O. ficus-indica*, not normally distributed data or data with unequal variances), one-way ANOVA (for *C. bigelovii*, normally distributed data with equal variances), or Kruskal-Wallis rank-sum test (for *C. bigelovii*, not normally distributed data or data with unequal variances). Due to the small sample size, the conservative Tukey’s test was applied as a post-hoc test. Data of a parameter were pooled if no significant differences were found between the plants of one species. To test for significant differences between $WE_{0}$ and $WE_{21}$, we employed tests for paired samples (paired t-test for normally distributed data with equal variances and Wilcoxon signed-rank test for not normally distributed data or data with unequal variances). One-sample t-test was used to test whether the bending stiffness ratios differed significantly from 1, and whether $HE_{21}$ differed significantly from 0 (all data were normally distributed). The Welch’s two-sample t-test and one-way ANOVA were used to test for differences of bending elastic modulus (all data were normally distributed). Levels of significance were: *p* > 0.05: not significant (n.s.); *p* ≤ 0.05: significant (*); *p* ≤ 0.01: very significant (**); *p* ≤ 0.001: highly significant (***).

## 5. Conclusions

Our combined morphological, anatomical, and biomechanical results gained in the context of self-healing after artificial ring cuts in two species of cactus suggest a very high selection pressure on the restoration of structural integrity in these plants in terms of the formation of the wound periderm as a protective transpiration-reducing layer. Protection against water loss and pathogen infection seems to play a more decisive role than the complete restoration of the mechanical integrity of branches for which the principle of “sufficient is good enough” seems to apply.

## Figures and Tables

**Table 1:** Mean values ± standard deviation (SD) of bending stiffness ratios in various states for *Opuntia ficus-indica* and *Cylindropuntia bigelovii*. These values quantify the stiffening (ratio > 1) or softening (ratio < 1) of the tissues between the first and second linear range.

	Bending Stiffness Ratio			
States	Unwounded	Wounded	Healed	All States ^1^
**Sample size (*N*)**	14	14	14	42
***O. ficus-indica***	0.88 ± 0.14	0.95 ± 0.21	0.95 ± 0.13	0.93 ± 0.17
***C. bigelovii***	1.16 ± 0.13	1.07 ± 0.17	1.18 ± 0.18	1.14 ± 0.17

Note: Bending stiffness ratios do not differ significantly between states for the two species. ^1^ The pooled data of all states significantly differ from one for *O. ficus-indica* (one-sample t-test, *t* = −2.92, df = 41, *p* = 0.005) and *C. bigelovii* (one-sample *t*-test, *t* = 5.19, df = 41, *p* < 0.001).

**Table 2:** Median values with respective interquartile ranges (IQR) of the mechanical parameters presented for the individual plants A and B of *O. ficus-indica* and plants C–E of *C. bigelovii*.

	*O. ficus-indica*		*C. bigelovii*		
	Plant A	Plant B	Plant C	Plant D	Plant E
**Sample size (*N*)**	8	6	5	4	5
**Bending stiffness first linear range (Nmm^2^)**					
**unwounded**	13,048 (7589)	17,978 (3541)	12,759 (1530)	16,588 (4637)	15,534 (3383)
**wounded**	9274 (6221)	13,470 (5669)	8873 (343)	11,619 (1036)	12,355 (502)
**healed**	8598 (2206)	9001 (4307)	10,423 (938)	12,303 (1384)	11,334 (690)
**Bending stiffness second linear range (Nmm^2^)**					
**unwounded**	10,548 (7755)	16,746 (7017)	16,715 (3664)	19,016 (1995)	18,804 (1685)
**wounded**	7863 (5932)	13,650 (6718)	9447 (2109)	11,963 (1308)	12,020 (506)
**healed**	7766 (2829)	7777 (4434)	11,356 (987)	13,509 (1577)	12,941 (942)
**Work (Nmm)**					
**unwounded**	7.4 (3.2)	11.6 (1.8)	8.6 (0.6)	10.6 (3.3)	10.0 (1.0)
**wounded**	5.9 (4.0)	8.5 (4.2)	6.1 (0.4)	7.5 (0.5)	7.8 (0.6)
**healed**	5.8 (1.5)	5.6 (1.5)	6.6 (0.6)	7.9 (1.4)	7.2 (0.6)

**Table 3:** Median values with respective interquartile ranges (IQR) of the healing effect $HE_{21}$ presented for the individual plants A and B of *O. ficus-indica* and C–E of *C. bigelovii*. Pooled data are given if values between the individual plants did not differ significantly. Significant differences from zero are marked by asterisks (one-sample t-test). * *p* ≤ 0.05, ** *p* ≤ 0.01, *** *p* ≤ 0.001.

	*O. ficus-indica*			*C. bigelovii*			
	Plant A	Plant B	Pooled Data	Plant C	Plant D	Plant E	Pooled Data
**Sample size (*N*)**	8	6	14	5	4	5	14
**$HE_{21}$ of bending stiffness first linear range (%)**							
	−15.22 (30.52)	−22.03 * (28.49)	−17.80 ** (21.06)	14.88 * (2.45)	−1.93 (20.83)	−6.70 (0.75)	2.44 (20.44)
**$HE_{21}$ of bending stiffness second linear range (%)**							
	−1.47 (25.88)	−31.21 *** (11.53)		12.32 * (8.74)	8.72 * (4.91)	9.48 * (2.90)	9.72 *** (7.04)
**$HE_{21}$ of work (%)**							
	1.59 (26.39)	−19.00 ** (10.68)	−14.96 * (27.16)	5.92 (2.76)	6.12 (9.78)	−5.44 (20.43)	5.30 (12.12)

## Supplementary Materials

Supplementary materials can be found at https://www.mdpi.com/1422-0067/21/13/4630/s1. Figure S1: Tangential sections of the wound area after artificial ring incision in *Opuntia ficus-indica* (**a**–**h**) and *Cylindropuntia bigelovii* (**i**–**p**), stained with phloroglucinol to establish the presence of lignin. The sections were microscopically examined directly after wounding (**a**,**i**), after a healing period of 1 day (**b**,**j**), 3 days (**c**,**k**), 7 days (**d**,**l**), 10 days (**e**,**m**), 14 days (**f**,**n**), 21 days (**g**,**o**), and 31 days (**h**,**p**). Scale bars = 500 µm. Table S1: Raw data of measurements carried out on *Opuntia ficus-indica* and *Cylindropuntia bigelovii*.

## Abbreviations

WE	Wounding effect
HE	Healing effect
SD	Standard deviation
IQR	Interquartile range
E	Bending elastic modulus
I	Axial second moment of area
RWC	Relative water content
STL	Stereolithography
CAD	Computer-aided design
COM	Center of mass
EI	Bending stiffness
uv	Unwounded value
wv	Wounded value

## Appendix A

**Table A1:** Statistics of mechanical parameters comparing the individual plants of the plant species *O. ficus-indica* and *C. bigelovii*.

Parameter Comparison	*O. ficus-indica* Plants A and B *n* = 14	*C. bigelovii* Plants C, D, and E *n* = 14
**BS 1 ^1^**		
$WE_{0}$—$WE_{0}\;$^2^	*t* = −0.441, df = 9.438, *p* = 0.669 (1) n.s.	Df = 2, Sum Sq = 293, Mean Sq = 146.5, F = 0.391, *p* = 0.685 (5) n.s.
$WE_{21}$—$WE_{21\;}$^3^	*t* = −2.647, df = 11.804, *p* = 0.0216 (1) *	Df = 2, Sum Sq = 350, Mean Sq = 175.2, F = 0.514, *p* = 0.612 (5) n.s.
$HE_{21}$—$HE_{21}$^4^	*t* = 1.519, df = 11.616, *p* = 0.155 (1) n.s.	Df = 2, Sum Sq = 1102, Mean Sq = 551.2, F = 4.36, *p* = 0.0403 Tukey’s test: all *p* > 0.055 (5,7) n.s.
**BS 2 ^5^**		
$WE_{0}$—$WE_{0}$	W = 32, *p* = 0.345 (3) n.s.	Df = 2, Sum Sq = 32.1, Mean Sq = 16.04, F = 0.162, *p* = 0.853 (5) n.s.
$WE_{21}$—$WE_{21}$	*t* = 1.832, df = 9.158, *p* = 0.0996 (1) n.s.	Df = 2, Sum Sq = 44.7, Mean Sq = 22.35, F = 0.228, *p* = 0.8 (5) n.s.
$HE_{21}$—$HE_{21}$	*t* = 3.652, df = 11.084, *p* = 0.00376 (1) **	Df = 2, Sum Sq = 5.59, Mean Sq = 2.796, F = 0.124, *p* = 0.885 (5) n.s.
**Work**		
$WE_{0}$—$WE_{0}$	*t* = 0.446, df = 10.945, *p* = 0.665 (1) n.s.	Df = 2, Sum Sq = 46.3, Mean Sq = 23.16, F = 0.085, *p* = 0.92 (5) n.s.
$WE_{21}$—$WE_{21}$	*t* = 3.343, df = 9.704, *p* = 0.00777 (1) **	χ^2^ = 2.143, df = 2, *p* = 0.343 (6) n.s.
$HE_{21}$—$HE_{21}$	*t* = 2.113, df = 11.653, *p* = 0.0569 (1) n.s.	Df = 2, Sum Sq = 306, Mean Sq = 153.0, F = 1.227, *p* = 0.33 (5) n.s.

^1^ BS 1 = Bending stiffness (based on first linear range of force-defection curves), ^2^ *WE*_0_ = wounding effect immediately after ring incision, ^3^ *WE*_21_ = wounding effect after a 21-day healing period, ^4^ *HE*_21_ = healing effect after a 21-day healing period ^5^ BS 2 = bending stiffness (based on second linear range of force-defection curves), (1) Welch’s two-sample t-test, (2) paired t-test, (3) Wilcoxon rank-sum test, (4) Wilcoxon signed-rank test, (5) ANOVA, (6) Kruskal-Wallis rank-sum test, (7) Tukey’s post hoc test. n.s. = not significant, * *p* ≤ 0.05, ** *p* ≤ 0.01, *** *p* ≤ 0.001.

**Table A2:** Statistics of mechanical parameters comparing wounding effects at two different stages of the two plant species *O. ficus-indica* and *C. bigelovii*.

Plant	BS 1 ^1^ $WE_{0}$${\;}^{2}—WE_{21}{\;}^{3}$	BS 2 ^4^ $WE_{0}$$—WE_{21}$	Work $WE_{0}$$—WE_{21}$
***O. ficus-indica***			
Plant A (*N* = 8)	*t* = 1.4159, df = 7, *p* = 0.1997 (2) n.s.	V = 16, *p* = 0.8438 (4) n.s.	*t* = −0.6040, df = 7, *p* = 0.565 (2) n.s.
Plant B (*N* = 6)	*t* = 3.5331, df = 5, *p* = 0.0167 (2) *	t = 7.2921, df = 5, *p* = 0.00076 (2) ***	*t* = −4.528, df = 5, *p* = 0.0062 (2) **
***C. bigelovii***			
Plant C (*n* = 5)	*t* = -3.0339, df = 4, *p* = 0.039 (2) *	*t* = −3.8951, df = 4, *p* = 0.0176 (2) *	V = 14, *p* = 0.125 (4) n.s.
Plant D (*n* = 4)	*t* = 0.58275, df = 3, *p* = 0.601 (2) n.s.	*t* = −4.9121, df = 3, *p* = 0.0162 (2) *	*t* = −0.1308, df = 3, *p* = 0.904 (2) n.s.
Plant E (*n* = 5)	*t* = 1.142, df = 4, *p* = 0.3172 (2) n.s.	*t* = −4.4061, df = 4, *p* = 0.0116 (2) *	*t* = 0.6218, df = 4, *p* = 0.5677 (2) n.s.

^1^ BS 1 = Bending stiffness (based on first linear range of force-defection curves), ^2^ *WE*_0_ = wounding effect immediately after ring incision, ^3^ *WE*_21_ = wounding effect after a 21-day healing period, ^4^ *HE*_21_ = healing effect after a 21-day healing period ^5^ BS 2 = bending stiffness (based on second linear range of force-defection curves), (1) Welch’s two-sample t-test, (2) paired t-test, (3) Wilcoxon rank sum test, (4) Wilcoxon signed-rank test, (5) ANOVA, (6) Kruskal-Wallis rank-sum test, (7) Tukey’s post hoc test. n.s. = not significant, * *p* ≤ 0.05, ** *p* ≤ 0.01, *** *p* ≤ 0.001.
