# Supplementary figures and images for: Self-Repair in Cacti Branches: Comparative Analyses of Their Morphology, Anatomy, and Biomechanics
Source: Int J Mol Sci. 2020 Jun 29;21(13):4630. doi: 10.3390/ijms21134630 (PMC7370035; doi:10.3390/ijms21134630)

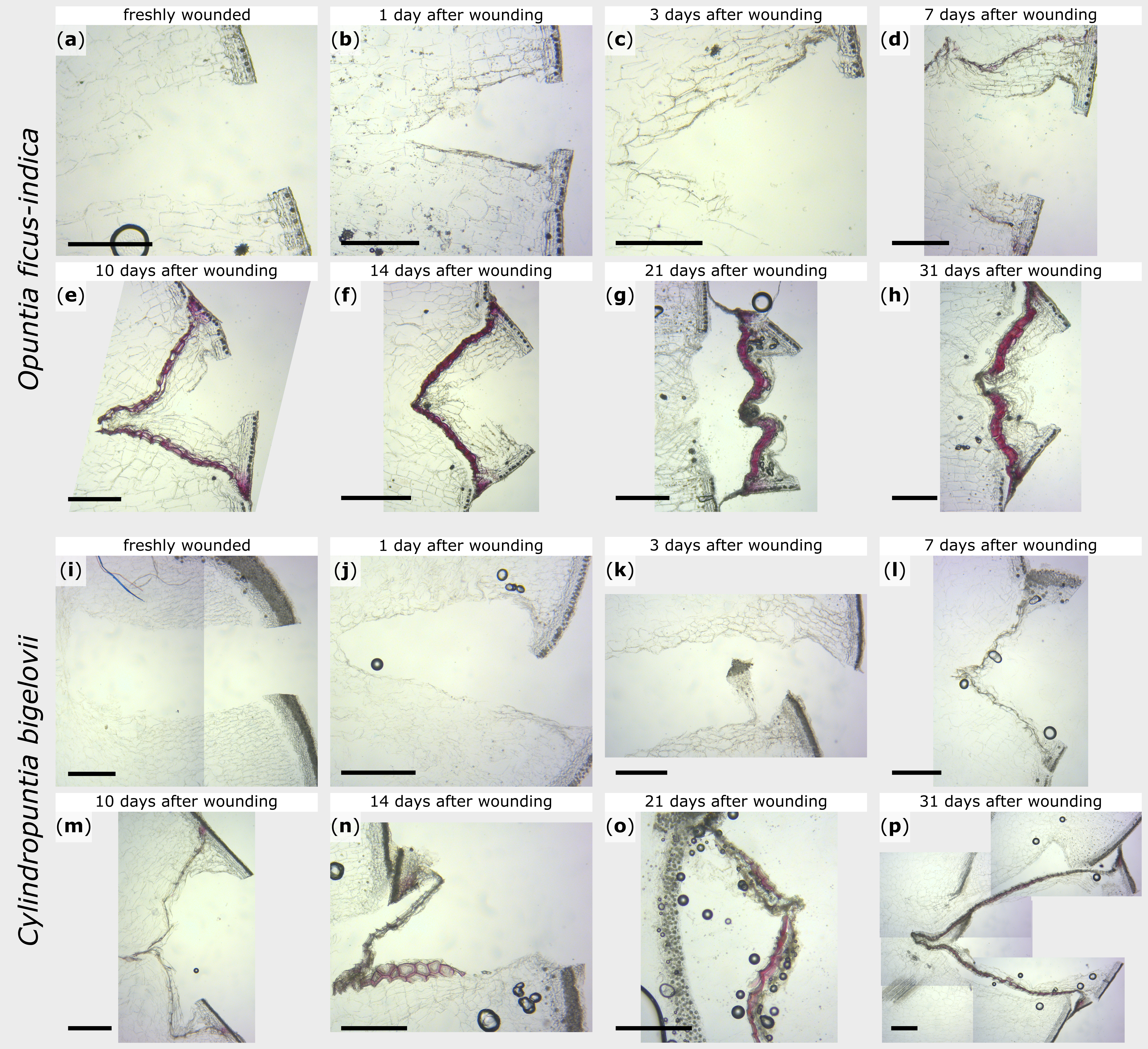

Supplement: Supplementary file 1 [file ijms-21-04630-s001.zip › Supplement Figure S1_Anatomy.png]
